# Effect of obstructive sleep apnea on right ventricular ejection fraction in patients with hypertrophic obstructive cardiomyopathy

**DOI:** 10.1002/clc.23429

**Published:** 2020-09-16

**Authors:** Shengwei Wang, Hao Cui, Keshan Ji, Changwei Ren, Hongchang Guo, Changsheng Zhu, Yongqiang Lai, Shuiyun Wang

**Affiliations:** ^1^ Department of Cardiovascular Surgery Center Beijing Anzhen Hospital, Capital Medical University, Beijing Institute of Heart, Lung and Blood Vascular Diseases Beijing China; ^2^ Department of Cardiovascular Surgery Mayo Clinic Rochester Minnesota USA; ^3^ Department of Special Medical Treatment Center, State Key Laboratory of Cardiovascular Disease, Fuwai Hospital, National Center for Cardiovascular Diseases Chinese Academy of Medical Sciences and Peking Union Medical College Beijing China; ^4^ Department of Cardiovascular Surgery, State Key Laboratory of Cardiovascular Disease, Fuwai Hospital, National Center for Cardiovascular Diseases Chinese Academy of Medical Sciences and Peking Union Medical College Beijing China

**Keywords:** hypertrophic obstructive cardiomyopathy, obstructive sleep apnea, right ventricular dysfunction, right ventricular ejection fraction

## Abstract

**Background:**

Obstructive sleep apnea (OSA) is a common disease associated with worse structural and functional impairment of the heart in patients with hypertrophic obstructive cardiomyopathy (HOCM).

**Hypothesis:**

The presence and severity of OSA can decrease the right ventricular ejection fraction (RVEF) in patients with HOCM.

**Methods:**

In total, 151 consecutive patients with a confirmed diagnosis of HOCM at Fuwai Hospital between September 2017 and September 2018 were included. Polysomnography and cardiac magnetic resonance imaging were performed in all patients.

**Results:**

Overall, 84 (55.6%) patients were diagnosed with OSA. The RVEF significantly decreased with the severity of OSA (none, mild, moderate‐severe: 46.1 ± 8.2 vs 42.9 ± 7.5 vs 41.4 ± 7.4, *P* = .009). The apnea‐hypopnea index (AHI) was significantly high in patients with RVEF<40% among the different OSA groups (mild, moderate:7.7 ± 2.4 vs 9.6 ± 2.9, *P* = .03; 24.4 ± 9.0 vs 36.3 ± 18.0, *P* = .01). In the multiple linear regression model, the right ventricular end‐systolic volume (β = −0.28, *P* < .001), AHI (β = −0.09, *P* = .02), and oxygen desaturation index (β = −0.11, *P* = .04) were independently associated with a decrease in RVEF (adjusted R^2^ = 0.347, *P* < .001). Furthermore, the prevalence of RVEF<40% was high in patients with OSA. Compared with RVEF>40%, RVEF<40% was associated with more symptoms, mainly chest pain, chest distress, NYHA class III or IV, pulmonary hypertension, and moderate or severe mitral regurgitation.

**Conclusion:**

In patients with HOCM, the presence and severity of OSA is independently associated with a lower RVEF. In addition, compared with patients with RVEF>40%, those with RVEF<40% had more symptoms, including chest pain, chest distress, and NYHA class III or IV.

## 1 INTRODUCTION

Hypertrophic cardiomyopathy (HCM) is the most common inherited heart disease with a prevalence of approximately 1:500 in the general population. About two‐thirds of patients with HCM present left ventricular outflow tract (LVOT) obstruction, which has been defined as hypertrophic obstructive cardiomyopathy (HOCM).^1^ Obstructive sleep apnea (OSA) is a common sleep disturbance in adults that has been increasingly implicated in the pathogenesis and complications of cardiovascular disease, including HCM.^2^, ^3^ Studies have reported that OSA is common in patients with HCM with a prevalence of 32% to 71%.^4^ OSA is independently associated with increased structural and functional cardiac impairment in patients with HCM, including higher NYHA functional classes, and a worse quality of life.^3^ Previous studies have revealed that OSA is independently associated with cardiac arrhythmias, enlargement of the left atrial and ascending aorta in patients with HCM.^5^, ^6^, ^7^ A previous study showed that OSA was associated with right ventricular dysfunction which was reflected by the right ventricular ejection fraction (RVEF), and it could be reversed by OSA treatment.^8^ In addition, a previous study reported that in HCM patients, right ventricular dysfunction is common and independently associated with an increased risk of death or transplantation.^9^ However, few studies have investigated the relationship between OSA and right ventricular dysfunction in HOCM, and factors associated with right ventricular dysfunction in these patients are unclear. Therefore, in the present study, we aimed to evaluate the factors associated with right ventricular dysfunction reflected by RVEF, and clinical correlates of lower RVEF in patients with HOCM.

## 2 METHODS

### 2.1 Study population

This was a prospective observational research study, which included 151 consecutive patients with HOCM who were referred to the Fuwai Hospital (Beijing, China) between September 2017 and September 2018. All patients in the present study underwent cardiac magnetic resonance imaging (MRI) and polysomnography (PSG). The inclusion criteria for patients in this study were the following: (a) age > 18 years; (b) agreement to participate in the study; and (c) diagnosis of HOCM. Patients were excluded if they had >50% of central respiratory events, incomplete sleep recording data, were younger than 18 years, had previous septal reduction therapy (septal myectomy or alcohol septal ablation), or a previous diagnosis of OSA. The diagnostic criteria of HOCM include an unexplained septal hypertrophy with a thickness of >15 mm and a LVOT gradient >50 mm Hg at rest or with provocation according to guidelines.^1^, ^10^ All patients in this study provided informed consent, including use of biomarker analysis and clinical data, prior to enrolment. The study was approved by the Ethics Committee of the Fuwai Hospital (Ethic committee study number: 2019‐1213) and was conducted in accordance with the ethical principles stated in the Declaration of Helsinki.

### 2.2 Polysomnography

Standard PSG (Embletta, Embla, UK) was performed in all patients. Respiratory events and data were scored by an experienced scorer. Obstructive apnea was defined as the absence of oro‐nasal airflow for at least 10 seconds in the presence of out‐of‐phase thoracoabdominal effort. Hypopnea was defined as a 50% reduction in oro‐nasal airflow for 10 seconds associated with a 4% decrease in oxygen saturation. OSA was classified based on the presence of thoracic efforts. The total recording time was used as the denominator to calculate the apnea‐hypopnea index (AHI). The total AHI was calculated and represented the number of events per hour of sleep. The AHI was calculated as the mean number of apneas and hypopneas per hour of sleep. Mild, and moderate to severe OSA were defined as an AHI ≥5 events/h and > 15 events/h, respectively.^11^

### 2.3 Imaging analysis

Echocardiographic evaluation was performed on patients by an experienced physician using an E9 ultrasound system. Diameters of the cardiac chambers were expressed as the maximum value of the anteroposterior diameter in cardiac cycles. The thicknesses of the interventricular septum and ventricular wall were determined during diastole. Aside from the maximum thickness, the representative thickness of the interventricular septal thickness (IVS) was also recorded to indicate overall thickness. The right ventricular diameter was measured in the apical four chamber view. The long axis of the parasternal pulmonary artery was used to measure the pulmonary artery diameter. The LVOT gradient was calculated using a simplified Bernoulli equation. Pulmonary hypertension was defined as pulmonary artery systolic pressure ≥ 35 mm Hg. Measurement of the right ventricular free wall thickness (RVWT) was performed at end‐diastole, below the tricuspid annulus at a distance approximating the length of the anterior tricuspid leaflet and RVWT >5 mm was used to define the presence of right ventricular hypertrophy.^12^, ^13^ The measurements of the left ventricular ejection fraction (LVEF) were determined by following the American Society of Echocardiography recommendations.^14^

Cardiac MRI was performed using a 1.5 Tesla cardiac magnetic scanner (Magnetom Avanto, Siemens Medical Solutions, Erlangen, Germany). Cine scans in the cardiac short‐axis and long‐axis views were acquired by applying true imaging with steady‐stage precession sequence (TrueFISP). Image analysis was performed using a commercial imaging workstation (Siemens Medical Systems). The LVEF and the indexed left ventricular mass and volume were measured by analyzing a short‐axis cine image. Inner and outer myocardial edges were manually delineated. LGE was determined semiautomatically as a percentage of the total myocardium and defined as an intensity >6 SD above the normal myocardium as indicated in previous studies.^15^ RVEF was measured by an independent investigator using volumetric measurements on cardiac MRI, and manual tracing of the endocardial borders of the contiguous short‐axis slices at end‐diastole and end‐systole stages allowed for the calculation of right ventricular end diastolic volume and right ventricular end‐systolic volume.^16^

### 2.4 Statistical analysis

The results in the present study are expressed as mean ± SD, median (interquartile range), or percentage, where appropriate. The *χ* ^2^ or Fisher's exact test was used to compare nominal variables, and differences among the three groups were compared using one‐way ANOVA. Before linear regression analysis, Spearman's rank correlation analysis was performed to select parameters which correlated with RVEF. Furthermore, multivariate linear regression models were used to analyze RVEF. Variables with P‐value <.10 on univariate analysis were entered into a multivariate analysis. All reported probability values were two‐tailed, and a P‐value <.05 was considered statistically significant. The SPSS version 24.0 software (IBM) and Prism GraphPad 7.0 (GraphPad Software Inc., La Jolla, CA, USA) were used for calculations and illustrations, respectively.

## 3 RESULTS

### 3.1 Baseline characteristics of the study population

We enrolled 151 patients in the present study (Figure S1). The baseline characteristics of the study population, as well as subgroups stratified according to the severity of OSA, are shown in Table 1. Patients with OSA were older, while a family history of HCM or sudden cardiac death was more common in patients without OSA. Furthermore, compared to patients without OSA, patients with OSA had higher levels of fasting blood glucose, big endothelin, high‐sensitivity C‐reactive protein (Hs‐CRP), and norepinephrine. In addition, patients with OSA had a higher incidence of syncope (11.9% vs 15.6% vs 33.3%, *P* = .02], and atrial fibrillation [9.0% vs 20.0% vs 25.6%, *P* = .02]). There were no differences in any of the other parameters detailed in Table 1.

**TABLE 1:** Baseline characteristics of the study population

Variable	No OSA (n = 67)	Mild OSA (n = 45)	Moderate‐severe OSA (n = 39)	*P* value
Age, y	43.7 ± 14.4	54.4 ± 11.2^a^	53.2 ± 11.7^a^	<.001
Male	41 (61.2%)	26 (57.8%)	26 (66.7%)	.81
Body mass index, kg/m^2^	24.2 ± 2.7	25.0 ± 3.1	25.3 ± 2.7	.15
Family history of HCM or SCD	18 (26.9%)	5 (11.1%)^a^	4 (10.3%)^a^	.02
NYHA III or IV	49 (73.1%)	32 (71.1%)	24 (61.5%)	.44
Fasting blood glucose, mmol/L	4.4 ± 0.7	4.8 ± 0.8^a^	5.2 ± 1.7^a^ ^,^ ^b^	<.001
Big endothelin, pmol/L	0.33 ± 0.13	0.50 ± 0.30^a^	0.57 ± 0.37^a^	<.001
Hs‐CRP, mg/L	1.42 (1.06‐1.85)	1.84 (0.60‐2.79)	2.01 (0.60‐3.65)^a^ ^,^ ^b^	.02
BNP, pg/mL	1400.0 (581.9‐2116.0)	1189.0 (521.8‐2707.0)	1351.0 (495.2‐1919.0)	.74
Norepinephrine, ng/ml	0.28 ± 0.05	0.31 ± 0.08^a^	0.35 ± 0.11^a^ ^,^ ^b^	<.001
Concomitant disease				
Hypertension	7 (10.4%)	11 (24.4%)	8 (20.5%)	.13
Hyperlipidemia	15 (22.4%)	11 (24.4%)	12 (30.8%)	.63
Diabetes mellitus	3 (4.5%)	3 (6.7%)	3 (7.7%)	.77
Clinical presentation				
Chest pain	17 (25.4%)	8 (17.8%)	23 (59.0%)	.87
Amaurosis	10 (15.2%)	7 (15.6%)	5 (12.8%)	.93
History of syncope	8 (11.9%)	7 (15.6%)	13 (33.3%)^a^ ^,^ ^b^	.02
Chest distress	37 (55.2%)	24 (53.3%)	23 (59.0%)	.87
Dizziness	11 (16.4%)	8 (17.8%)	4 (10.5%)	.62
Palpitation	8 (11.9%)	4 (8.9%)	9 (23.1%)	.14
Atrial fibrillation	6 (9.0%)	9 (20.0%)^a^	10 (25.6%)^a^ ^,^ ^b^	.02
Medical therapy				
Beta‐blockers	44 (65.7%)	34 (75.6%)	26 (66.7%)	.51
Calcium‐channel blockers	10 (14.9%)	8 (17.8%)	3 (7.7%)	.39

Abbreviations: BNP, brain natriuretic peptide; HCM, hypertrophic cardiomyopathy; Hs‐CRP, high‐sensitivity C‐reactive protein; NYHA, New York Heart Association; OSA, obstructive sleep apnea; SCD, sudden cardiac death.

^a^ *P* < .05, Mild OSA or moderate‐severe OSA vs no OSA.

^b^ *P* < .05, Mild OSA vs moderate‐severe OSA.

### 3.2 Imaging parameters of the study population

**FIGURE 1:**
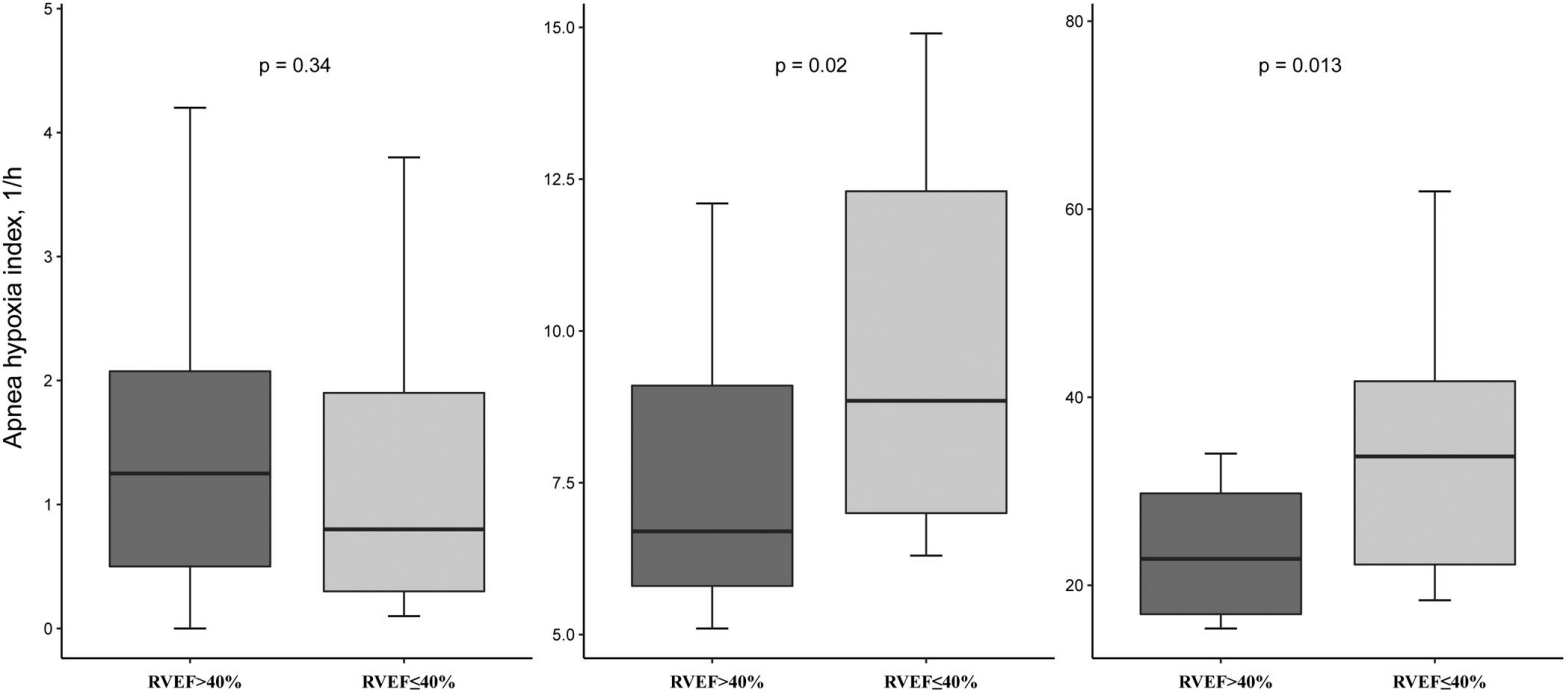
The value of apnea hypopnea index according to the level of RVEF among the different OSA groups: A, No OSA; B, Mild OSA; and C, Moderate‐severe OSA. OSA, obstructive sleep apnea; RVEF, right ventricular ejection fraction

The imaging parameters of the study population stratified according to the severity of OSA are shown in Table 2. As also shown in Table 2, the echocardiographic indices, including the prevalence of pulmonary hypertension, right ventricular hypertrophy, moderate or severe mitral regurgitation, left atrial diameter, left ventricular end‐diastolic dimension, pulmonary artery diameter, right ventricular diameter, and right ventricular wall thickness, were significantly increased with the severity of OSA. In addition, we found that the value of the indexed left ventricular mass, indexed left ventricular volume, and LGE% were also increased with the severity of OSA. Most importantly, the RVEF (46.1 ± 8.2 vs 42.9 ± 7.5 vs 41.4 ± 7.4, *P* = .009) was decreased with the severity of OSA. The prevalence of RVEF<40% was also significantly higher in patients with OSA. In addition, the AHI value was significantly higher in patients with RVEF<40% compared to the different OSA groups (Figure 1).

**TABLE 2:** Imaging parameters of the study population

Variable	No OSA (n = 67)	Mild OSA (n = 45)	Moderate‐severe OSA (n = 39)	*P* value
Echocardiographic indices				
LVEF, %	67.9 ± 6.0	67.8 ± 5.7	68.1 ± 6.4	.97
Left atrial diameter, mm	42.9 ± 6.7	44.9 ± 5.9	46.0 ± 7.7	.06
LVEDD, mm	40.5 ± 3.9	42.6 ± 4.6^a^	44.8 ± 4.4^[Link]^, ^[Link]^	<.001
IVST, mm	21.6 ± 5.6	20.8 ± 4.1	20.4 ± 2.6	.45
RVH	33 (49.3%)	25 (55.6%)	29 (74.4%)^[Link]^, ^[Link]^	.02
RVWT, mm	5.9 ± 1.5	6.3 ± 1.7	7.2 ± 1.6^[Link]^, ^[Link]^	.001
Pulmonary artery diameter, mm	23.0 ± 2.9	24.5 ± 4.0^a^	24.8 ± 2.6^a^	.01
Right ventricular diameter, mm	21.1 ± 2.5	21.9 ± 3.0	22.6 ± 3.9^a^	.04
LVOT gradient, mm Hg	69.7 ± 24.4	72.5 ± 21.3	75.2 ± 25.6	.51
Pulmonary hypertension	2 (3.0%)	5 (11.1%)^a^	7 (17.9%)^[Link]^, ^[Link]^	.03
Moderate or severe MR	25 (37.3%)	21 (46.7%)	27 (69.2%)^[Link]^, ^[Link]^	.006
Cardiac magnetic resonance imaging				
Indexed LV mass, g/m^2^	93.9 ± 27.1	90.5 ± 23.9	107.0 ± 43.7^[Link]^, ^[Link]^	.04
Indexed LV volume, ml/m^2^	88.6 ± 28.9	86.5 ± 23.0	101.9 ± 41.6^a^	.05
LGE, % of LV mass	6.1 (3.4‐10.0)	6.8 (3.6‐12.9)	10.9 (8.3‐15.5)^[Link]^, ^[Link]^	.01
RVEDV, mL	78.6 ± 19.2	79.2 ± 27.2	80.4 ± 18.2	.92
RVESV, mL	42.5 ± 13.8	45.8 ± 21.3	46.9 ± 11.9	.35
RVEF, %	46.1 ± 8.2*	42.9 ± 7.5	41.4 ± 7.4	.009
RVEF<40%	13 (19.4%)	14 (31.1%)^a^	17 (43.6%)^[Link]^, ^[Link]^	.008

Abbreviations: IVST, interventricular septal thickness; LGE, late gadolinium enhancement; LVEDD, left ventricular end‐diastolic dimension; LVEF, left ventricular ejection fraction; LVOT, left ventricular outflow tract; OSA, obstructive sleep apnea; RVEDV, right ventricular end‐diastolic volume; RVEF, right ventricular ejection fraction; RVESV, right ventricular end‐systolic volume; RVH, right ventricular hypertrophy; RVWT, right ventricular wall thickness.

^a^P< .05, Mild OSA or moderate‐severe OSA vs no OSA.

^b^P < .05, Mild OSA vs moderate‐severe OSA.

### 3.3 PSG parameters of the study population

The PSG parameters of the study population are summarized in Table 3. Compared with patients without OSA, almost all PSG parameters, including the AHI, OSA index, oxygen desaturation index (ODI), and time ratio of SpO_2_ < 90% were significantly higher in patients with OSA. However, sleep efficiency, and REM sleep were significantly lower in patients with OSA. Importantly, there was no difference in the total sleep time among the three groups. Detailed information is shown in Table 3.

**TABLE 3:** Polysomnography parameters of the study population

Variable	No OSA (n = 67)	Mild OSA (n = 45)	Moderate‐severe OSA (n = 39)	*P* value
AHI, 1/h	1.2 (0.4‐2.1)	7.6 (6.0‐11.1)^a^	24.4 (18.5‐34.0)^a^ ^,^ ^b^	<.001
ODI, 1/h	2.6 (1.0‐6.3)	8.6 (6.5‐11.1)^a^	23.9 (16.8‐33.1)^a^ ^,^ ^b^	<.001
Mean SPO_2_ decline degree, %	4.8 ± 1.3	5.1 ± 1.2^a^	6.8 ± 2.0^a^ ^,^ ^b^	<.001
Time ratio of SpO_2_ < 90%, %	0.7 (0.0‐12.9)	1.4 (0.5‐7.4)^a^	13.7 (7.8‐39.2)^a^ ^,^ ^b^	.01
Sleep efficiency, %	69.9 ± 5.2	67.2 ± 5.0^a^	64.7 ± 3.2^a^ ^,^ ^b^	<.001
Sleep stage				
N1 sleep, %	15.8 ± 1.5	15.4 ± 2.1	14.9 ± 3.6^a^	.14
N2 sleep, %	65.8 ± 4.1	64.9 ± 3.2	66.1 ± 2.9	.11
N3 sleep, %	2.3 ± 1.4	1.8 ± 1.2	2.1 ± 1.3	.25
REM sleep, %	15.8 ± 1.6	15.4 ± 0.9	14.9 ± 1.6	.008
Total sleep time, min	529.7 ± 99.1	527.4 ± 108.6	510.7 ± 80.8	.61

*Note:* Values are presented as percentage, mean ± SD, or median (interquartile range) when appropriate.

Abbreviations: AHI, apnea hypopnea index; CAI, central apnea index; OAI, obstructive apnea index; ODI, oxygen desaturation index; OSA, obstructive sleep apnea.

^a^ *P* < 0.05, Mild OSA or moderate–severe OSA vs no OSA.

^b^ *P* < 0.05, Mild OSA vs moderate‐severe OSA.

### 3.4 Factors associated with RVEF and the effect of lower RVEF on HOCM patients

Multiple variables, including baseline characteristics, imaging parameters, and PSG parameters, were included in a correlation analysis to further identify the determinants of the decrease of RVEF in HOCM patients (Table 4). In the multiple linear regression analysis, right ventricular end‐diastolic volume (β = −0.28, *P* < .001), AHI (β = −0.09, *P* = .02), and ODI (β = −0.11, *P* = .04) were independently associated with a decrease in RVEF (adjusted R^2^ = 0.347, *P* < .001), when adjusted for age, sex, body mass index (BMI), and the other parameters that associated with a decrease of RVEF in the spearman analysis.

**TABLE 4:** Multiple linger regression between RVEF with significant variables from Spearman analysis

Variable	Univariate		Multivariate adjusted R^2^ = 0.347	
	rs	*P*	β	*P*
Age	−0.07	.39		
Sex	0.16	.06		
Body mass index	0.05	.51		
LVEDD	−0.17	.04		
Pulmonary artery diameter	−0.14	0.09		
Right ventricular diameter	−0.12	.05		
RVESV	−0.50	<.001	−0.28	<.001
RVWT	−0.176	.03		
LGE	−0.25	.002		
Apnea hypoxia index	−0.27	.001	−0.16	.023

Abbreviations: LGE, late gadolinium enhancement; LVEDD, left ventricular end‐diastolic dimension; RVEF, right ventricular ejection fraction; RVESV, right ventricular end‐diastolic volume; RVWT, right ventricular wall thickness.

The baseline characteristics according to the subgroup of RVEF are shown in Table S1. We found that HOCM patients with RVEF <40% had more symptoms, including chest pain, chest distress, NYHA class III or IV, and had a higher prevalence of pulmonary hypertension, and moderate or severe mitral regurgitation. In addition, the left ventricular end‐diastolic diameter, IVS, right ventricular diameter, right ventricular end‐diastolic volume, and right ventricular wall thickness were also significantly increased in patients with RVEF <40%. Furthermore, compared with a RVEF ≥40%, the prevalence of mild and moderate‐severe OSA was significantly higher in patients with a RVEF<40% (Figure S2).

## 4 DISCUSSION

To our knowledge, this is the first study to systematically investigate the relationship between OSA and RVEF in patients with HOCM. The main findings of the study were as follows: First, RVEF significantly decreased as the severity of OSA increased, and the AHI was significantly higher in patients with a RVEF<40% across the different OSA groups. Second, the AHI and ODI were independently associated with a decrease in RVEF after adjustment for other variables. Third, 29.1% of patients with HOCM had a lower RVEF (<40%), and it was associated with more symptoms, including chest pain, chest distress, NYHA class III or IV, a higher prevalence of pulmonary hypertension, and moderate or severe mitral regurgitation.

OSA may affect HOCM through many mechanisms, including activation of the sympathetic nervous system, an increase in insulin resistance, increased myocardial wall stress, and impairment of vascular endothelial function.^17^ The effect of OSA on the level of fasting blood glucose, endothelin, norepinephrine, and hs‐CRP have been well characterized in our previous studies.^18^ In addition, insulin resistance may increase the prevalence of atrial fibrillation in HOCM patients through its association with increased left atrial size or left ventricular diastolic impairment. Furthermore, our previous study also found that type 2 diabetes mellitus which is closely associated with insulin resistance is associated with a higher sudden cardiac death rate in a matched cohort with HOCM who underwent septal myectomy. However, there is a paucity of information on the relationship between OSA and right ventricular function in patients with HOCM. A previous study showed that right ventricular dysfunction reflected by RVEF is common in OSA, and it is reversible with the treatment of OSA.^8^ Another study published recently also showed that nocturnal hypoxemia is associated with right ventricle diastolic function in OSA.^19^ Furthermore, OSA is also an independent risk factor for subclinical right ventricular dysfunction in children.^19^ In our study, we found that the diameter of the pulmonary artery and right ventricle significantly increased with the severity of OSA, which might underly the right ventricular dysfunction. In addition, previous studies have reported that a RVEF <40% is a marker of increased risk of death and hospitalization, and a RVEF <20% is an independent risk factor for death and heart failure hospitalization in heart failure patients.^20^ Importantly, a recent study reported that more than half of HCM patients had right ventricular diastolic and systolic dysfunction, and right ventricular function was associated with exercise capacity.^21^ Furthermore, right ventricular hypertrophy correlated with increased calculated sudden cardiac death risk score and independently related to the presence of ventricular arrhythmias.^22^ In the present study, we found that RVEF <40% was more common in patients with OSA, and the value of RVEF significantly decreased with the severity of OSA. After adjustment for age, sex, and other relevant variables identified by the Spearman's correlation analysis, the AHI and ODI were also independently associated with a decrease in RVEF. These results suggest that OSA is an independent risk factor for the decrease in RVEF; however, the exact mechanism is unclear. In addition, right ventricular wall thickness increased with the severity of OSA, and right ventricular hypertrophy was more common in patients with OSA. The findings of the present study suggest that the presence and severity of OSA might cause impairment in right ventricular function in HOCM patients.

Previous studies have revealed that RVEF is influenced by right ventricular contractility, right ventricular end‐diastolic pressure, and pulmonary vascular pressure.^23^, ^24^ Experimental studies on mice have showed that intermittent hypoxia, which simulates the effect of repeated nocturnal apneas, can induce repeated instantaneous episodes of right ventricular overload through hypoxic vasoconstriction of pulmonary microcirculation. With time, this could lead to the remodeling of the pulmonary artery and chronic pulmonary hypertension.^25^, ^26^ In the present study, the prevalence of pulmonary hypertension was significantly higher in patients with OSA or RVEF<40%, which is consistent with previous studies. In addition, the prevalence of moderate or severe mitral regurgitation was also higher in patients with OSA. Mitral regurgitation, which influences pulmonary venous pressure, may be a determinant of right ventricular function, and several studies have suggested that mitral regurgitation severity is associated with a decrease in RVEF.^27^, ^28^ Furthermore, in our study, septal thickness was significantly higher in patients with a RVEF <40%, which is consistent with previous studies indicating RVEF may also be impaired because of ventricular interdependence associated with septal dysfunction and limited pericardial flexibility.^29^, ^30^

Another finding in our study was that a RVEF<40% was associated with symptoms, including chest pain, chest distress, and NYHA class III or IV in patients with HOCM. This is consistent with a previous study that found that right ventricular dysfunction helped to predict severe symptomatic HCM with high sensitivity and specificity.^31^ In addition, right ventricular dysfunction was independently associated with reduced exercise capacity in patients with HCM.^21^ In the present study, the presence and severity of OSA were independent risk factors for a decrease in RVEF, which affected the clinical manifestation of HCM patients.

Impairment of right ventricular function is associated with adverse clinical outcomes in cardiovascular disease, including chronic heart failure, right ventricular myocardial infarction, and ischemic and nonischemic cardiomyopathy.^30^ In addition, patients with severe untreated OSA had a higher incidence of fatal and nonfatal cardiovascular events than those without OSA, but the exact mechanism is unknown. In HOCM patients with OSA, a lower RVEF caused by OSA might be associated with a poor clinical outcome for these patients. Importantly, previous studies have reported that treatment of OSA can reverse right ventricular function, and may lead to a marked improvement in symptoms and reduce the LVOT gradient which would abrogate the need for septal reduction in patients with HOCM and OSA.^8^, ^32^ Therefore, we believe that early diagnosis and timely treatment for these patients may alleviate the symptoms caused by lower RVEF and improve the quality of life for these patients.

## 5 LIMITATIONS

There are some limitations in our study. First, sleep was only studied for one night in all the patients. In fact, the parameters of OSA showed a considerable night‐to‐night variability; thus, a single‐night diagnostic sleep study is prone to mis‐categorize the severity of OSA. Second, the present study was carried out in a single research center and the number of patients included was small. A multicenter study with a larger population of HOCM patients is needed to confirm our results, and a multicenter collaborative clinical trial studying the treatment effects of OSA is necessary. Finally, due to the cross‐sectional nature of the present study, it was not possible to conclude that OSA and a lower RVEF were independently associated with future adverse cardiovascular complications in patients with HOCM.

## 6 CONCLUSIONS

In patients with HOCM, the presence and severity of OSA was independently associated with a lower RVEF. In addition, patients with a RVEF <40% had symptoms including chest pain, chest distress, NYHA class III or IV, a higher prevalence of pulmonary hypertension, and moderate or severe mitral regurgitation. In addition, the left ventricular end‐diastolic diameter was also significantly increased in patients with a RVEF <40%, which is a marker for disease severity in HOCM. Further studies are needed to investigate the impact of OSA and RVEF on future cardiovascular complications as well as the safety and efficacy of OSA treatment in these patients.

## CONFLICT OF INTEREST

The authors declare no potential conflict of interest.

## Supporting information


**Figure S1** Flow diagram of study patients.

**Figure S2** Prevalence of obstructive sleep apnea in patients across different groups stratified by the level of RVEF.
RVEF = right ventricular ejection fraction.

**Table S1** Effect of lower RVEF on HOCM patients
