# Supplementary figures and images for: Effect of obstructive sleep apnea on right ventricular ejection fraction in patients with hypertrophic obstructive cardiomyopathy
Source: Clin Cardiol. 2020 Sep 16;43(10):1186–93. doi: 10.1002/clc.23429 (PMC7534009; doi:10.1002/clc.23429)

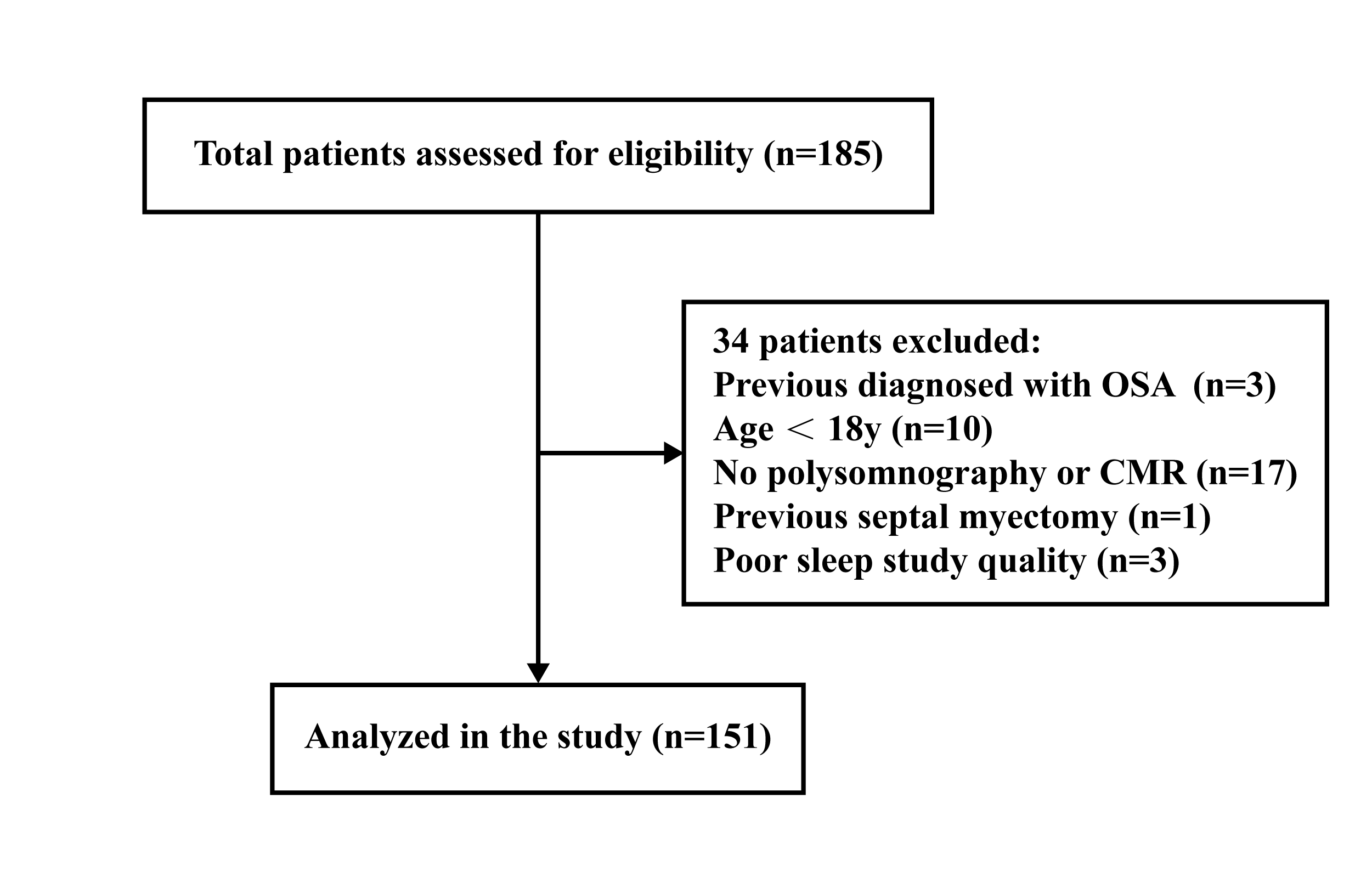

Supplement: Supplementary file 1 — Figure S1 Flow diagram of study patients. [file CLC-43-1186-s001.tif]

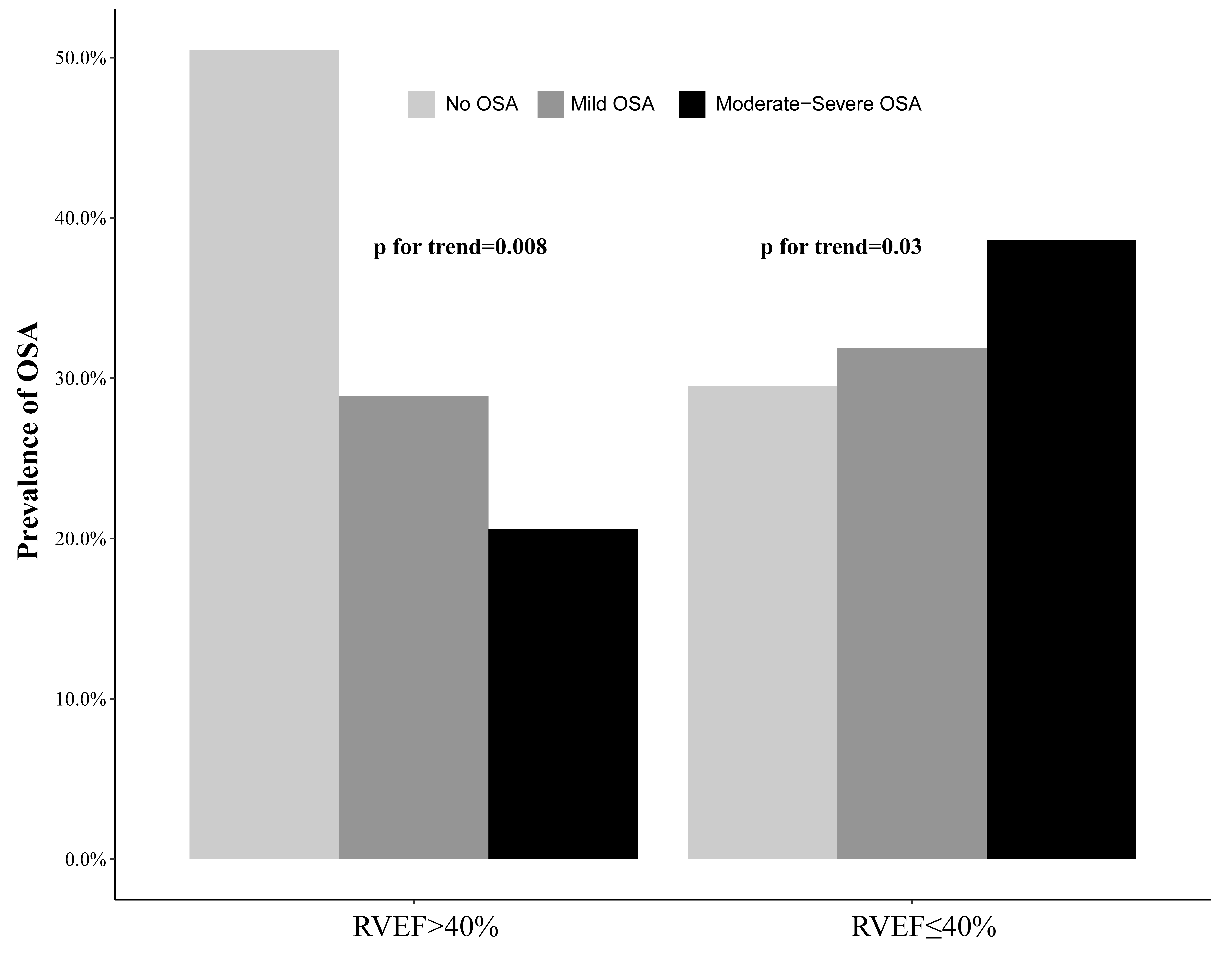

Supplement: Supplementary file 2 — Figure S2 Prevalence of obstructive sleep apnea in patients across different groups stratified by the level of RVEF. RVEF = right ventricular ejection fraction. [file CLC-43-1186-s002.tif]
