# Supplementary material for: Effect of obstructive sleep apnea on right ventricular ejection fraction in patients with hypertrophic obstructive cardiomyopathy
Source: Clin Cardiol. 2020 Sep 16;43(10):1186–93. doi: 10.1002/clc.23429 (PMC7534009; doi:10.1002/clc.23429)
Supplement: Supplementary file 3 — Table S1 Effect of lower RVEF on HOCM patients [file CLC-43-1186-s003.docx]

**Table S1** Effect of lower RVEF on HOCM patients

| Variable | RVEF<40%  (n=44） | RVEF≥40%  (n=107) | p Value |
| --- | --- | --- | --- |
| LVEDD, mm | 43.5±4.7 | 41.7±4.4 | 0.03 |
| IVST, mm | 21.5±4.6 | 19.8±4.9 | 0.04 |
| RVWT, mm | 6.9±1.7 | 6.2±1.6 | 0.03 |
| Right ventricular diameter, mm | 22.6±3.8 | 21.4±2.7 | 0.03 |
| RVESV, ml | 53.2±22.3 | 41.1±10.9 | <0.001 |
| Pulmonary hypertension | 8 (18.2%) | 6 (5.6%) | 0.02 |
| Moderate or severe MR | 28 (63.6%) | 45 (42.1%) | 0.02 |
| NYHA Ⅲ or Ⅳ | 36 (81.8%) | 69 (64.5%) | 0.04 |
| Chest pain | 17 (38.6%) | 23 (21.5%) | 0.03 |
| Chest distress | 30 (68.2%) | 54 (50.5%) | 0.04 |

Values are presented as percentage, mean ± SD, or median (interquartile range) when appropriate.

HOCM=hypertrophic obstructive cardiomyopathy; LVEDD=left ventricular end-diastolic dimension; IVST=interventricular septal thickness; RVESV=right ventricular end-diastolic volume; NYHA=New York Heart Association; RVEF=right ventricular ejection fraction.
